# Multimodal Navigation and Virtual Companion System: A Wearable Device Assisting Blind People in Independent Travel

**DOI:** 10.3390/s25134223

**Published:** 2025-07-06

**Authors:** Jingjing Xu, Caiyi Wang, Yancheng Li, Xuantuo Huang, Meina Zhao, Zhuoqun Shen, Yiding Liu, Yuxin Wan, Fengrong Sun, Jianhua Zhang, Shengyong Xu

**Affiliations:** 1 School of Integrated Circuits, Shandong University, Jinan 250010, China; 202332369@mail.sdu.edu.cn (C.W.); shenzq@mail.sdu.edu.cn (Z.S.); sunfr@sdu.edu.cn (F.S.); 2 School of Electronics, Peking University, Beijing 100871, China; lycheng@pku.edu.cn (Y.L.); xuantuo@pku.edu.cn (X.H.); 2301213327@pku.edu.cn (Y.L.); 2000012732@stu.pku.edu.cn (Y.W.); 3 School of Computer Science and Engineering, Tianjin University of Technology, Tianjin 300382, China; meinzhao@163.com (M.Z.); zjh@ieee.org (J.Z.)

**Keywords:** remote companion system, independent travel, navigation, obstacle voidance, wearable systems

## Abstract

Visual impairment or even loss seriously affects quality of life. Benefited by the rapid development of sound/laser detection, Global Positioning System (GPS)/Beidou positioning, machine vision and other technologies, the quality of life of blind people is expected to be improved through visual substitution technology. The existing visual substitution devices still have limitations in terms of safety, robustness, and ease of operation. The remote companion system developed here fully utilizes multimodal navigation and remote communication technologies, and the positioning and interaction functions of commercial mobile phones. Together with the accumulated judgment of backend personnel, it can provide real-time, safe, and reliable navigation services for blind people, helping them complete daily activities such as independent travel, circulation, and shopping. The practical results show that the system not only has strong operability and is easy to use, but also can provide users with a strong sense of security and companionship, making it suitable for promotion. In the future, this system can also be promoted for other vulnerable groups such as the elderly.

## 1. Introduction

According to the International Agency for the Prevention of Blindness (IAPB), approximately 1.1 billion people worldwide are affected by visual impairments, including around 43 million who are completely blind and about 295 million with moderate-to-severe vision impairment [1]. It is projected that the number of people with vision loss will increase by 600 million over the next 30 years [1]. For humans, approximately 80–90% of external information is acquired through vision, and impaired or even lost vision seriously affects quality of life. Consequently, the demand for visual restoration and/or assistive technologies and devices continues to persist, with expectations of even greater and more widespread needs in the future.

The two major technical approaches to solving the problem of normal travel for visually impaired patients are visual restoration and visual substitution [2,3]. Visual restoration involves the use of implanted visual prostheses, which provide an alternative solution or pathway for the impaired segments in visual information reception and transmission. This assists patients in reconstructing limited visual abilities to perceive partial image information, allowing them to make image judgments independently. This technological approach enables patients to form visual images on their own. Several artificial retinas have been tested in clinical applications for a small group of blinded people. However, these devices only had a total pixel of 60–2000 [2,4,5], which was not sufficient to offer clear images of visual subjects, such as details of a face. Due to high cost, surgical safety risks, and low visual resolution, the acceptance of this approach is limited, making widespread adoption challenging.

Scientists have long attempted to implant electrode arrays on the visual cortex of the brain to elicit visual responses in subjects. It was not until May 2020 that a groundbreaking study was published by the team led by Daniel Yoshor at Baylor College of Medicine [6]. This study demonstrated the ability to precisely present designated “visual images” in the minds of subjects through a series of sequentially timed dynamic electrical stimulation processes using an array of electrodes. However, it takes a while for subjects to respond to a dynamic electrode stimulation pattern and form “vision”. It remains to be determined whether this time can be compressed into less than 100 ms to accommodate the real-time responses required for daily activities.

In contrast, the technological approach of visual substitution seems more friendly for individuals with visual impairments due to its non-invasiveness. This approach utilizes technical means to analyze environmental image information, directly converting it into instructive signals guiding the actions of the subjects. In recent years, with the maturation of technologies such as ultrasound/laser detection [7], GPS/Beidou positioning [8], and machine vision [9,10], an increasing number of visual substitution technologies, such as electronic travel aids (ETAs) [11,12], electronic orientation aids (EOAs) [13], and positioning devices (PLDs) [14], have been developed. These devices have helped many visually disabled people, but they have not been widely promoted due to the limitations in terms of practicality. Because the implementation of practicality for artificial vision systems requires addressing two critical considerations [15]. Firstly, the selection of the technological pathway involves defining the type of interaction interface, interaction information, and interaction methods between the device and the user. Secondly, gaining the wearer’s trust is crucial. Up to now, there seems no widely used assistive system that instills confidence in individuals with visual impairments regarding the reliability, robustness, and overall performance of the device.

We have developed a multimodal navigation and virtual companion system (MNVCS) aimed at helping the blind, which includes basic components such as frontend hardware, mobile applications, human–machine interface, and command protocols. To address the two key issues mentioned above, the system has established a human–computer interaction mode with visual–tactile conversion as the main aim and auditory conversion as the auxiliary aim, providing efficient navigation and obstacle avoidance services for visually impaired patients in poor traffic scenarios without hindering their auditory alertness. Moreover, the introduction of remote virtual companionship function greatly enhances users’ trust in the system.

## 2. System Structure

The developed remote navigation system emphasizes both physical and psychological safety. The former is supported by mature and complementary input-feedback multimodal navigation technology, while the latter depends on remotely backed agents to achieve the following functions.

(1) Enhancing users’ security: remote navigation and companionship can be achieved by collaborating with frontend hardware and backend software systems.
(2) Tactile feedback is the main approach, with auditory feedback as a supplement. This is to avoid interfering with the user’s ability to listen to their environment, improving users’ ability to cope with multi-scenario stress feedback.
(3) There is no need to hold hands, freeing the user’s hands. In addition, the device should be simple, and possible to operate independently after simple training, with strong robustness.

**Figure 1:**
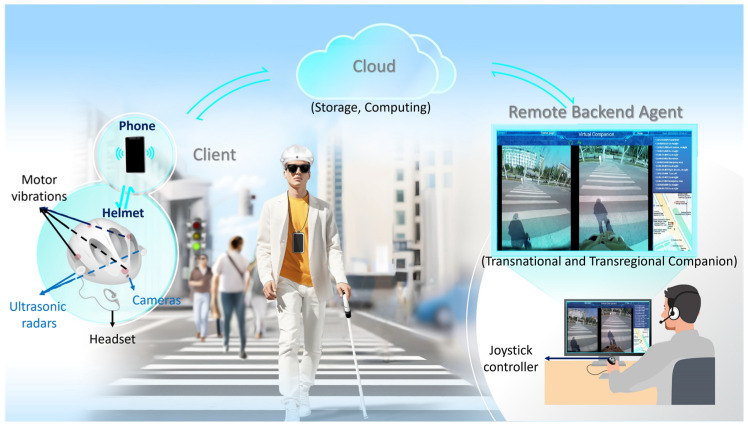
The overview of the developed multimodal navigation and virtual companion system.

The entire system is illustrated in Figure 1. The system includes the client, who is equipped with a wearable helmet and a phone installed with the self-developed application (app) operating on an Android system, the cloud as a server for information computing and storage, and the remote backend agent. The remote backend agent is the part of the service that allows personnel to understand users’ needs, obtain information about the surrounding environment, and then provide navigation and environmental information to users.

### 2.1. Hardware

The hardware used by the client mainly includes a wearable helmet and a mobile phone, as shown in Figure 1.

The wearable helmet is worn by users, and is equipped with input devices (sensors), a controller, and output devices (actuators). The input devices include sensors such as dual cameras (model: OV5640 160°; manufacturer: Renheng, Shenzhen, China) and ultrasonic radar (model: MZ-CS4004MK; manufacturer: Qizhou Electronics, Shenzhen, China) to obtain the image and distance information of the external environment. The image information is transmitted to the remote backend agent for environmental assessment, and the ultrasonic data is read by the local controller to determine if there are any obstacles near the front. The main control chip of the controller is ESP32 (model: ESP32-WROOM-32-N4; manufacturer: Espressif Systems, Shenzhen, China), which is linked to the mobile app via low-power Bluetooth. The controller reads ultrasonic data via serial port. Upon detecting obstacles within range, it triggers haptic feedback via motors and/or voice warnings through the headset. The controller also sends the collected ultrasound and image data to the mobile app via Bluetooth and uploads them to the cloud for storage. In addition, the controller needs to receive action instructions from remote backend agents and control the execution of actuators. The actuators include four evenly distributed motors (model: LCM1027A2445F; manufacturer: Leader Micro Electronics, Huizhou, China) that can vibrate to trigger touch and one headphone (model: Patriot, A220; manufacturer: Songang, Weifang, China) to guide the user’s behaviors. Upon receiving instructions from the agent, the mobile app sends a series of motor vibration control events to the controller in a queued manner. The controller, based on these events, controls the vibration of the motors, thereby triggering specific tactile stimuli in the user’s corresponding brain area, and guiding them to execute obstacle avoidance behaviors such as going straight, turning left or right, or stopping. The headphone is another medium for remote interaction between users and backend agents, which is mainly used to issue obstacle avoidance reminders and/or communication companionship needs in emergency situations. It should be noted that the set of single-sided headphone and the principle of not using them unless necessary are adopted, with the aim of minimizing interference with visually impaired patients’ auditory monitoring needs in relation to the outside world.

**Figure 2:**
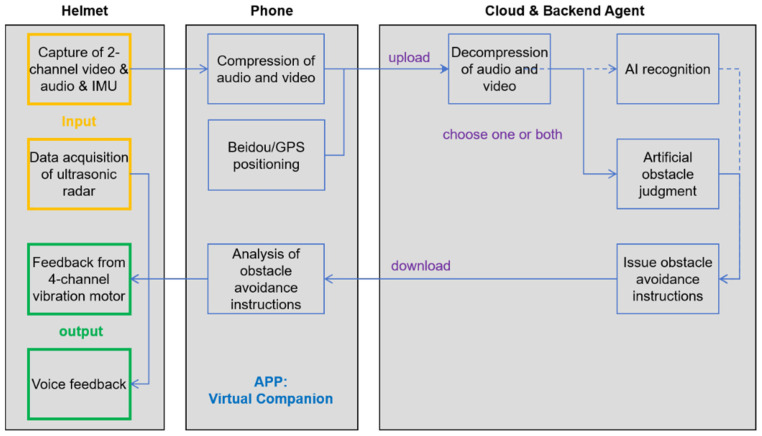
Hardware composition and data flow of the MNVCS.

The phone is equipped with a self-developed application that enables users to make reservations for travel services. This research-specific app serves as a centralized intermediary with capabilities for information exchange, decoding, and positioning functions. Figure 2 illustrates the data flow between hardware in MNVCS.

### 2.2. Software

**Figure 3:**
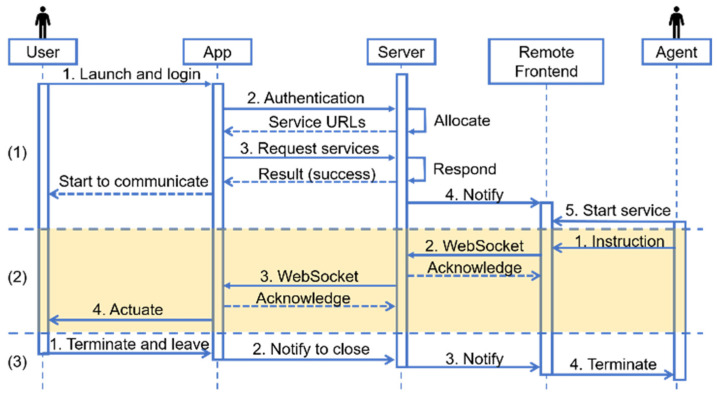
Sequence diagram of the mobile app, server, and agent frontend.

In MNVCS, the mobile app, server, and the backend used by agents were required to implement complex business logic. Figure 3 is the sequence diagram that describes the temporal logic among the mobile app, server, and agent frontend during the three representative processes of user service request and termination. (1) The user launched the app to log in. The authentication service on the server allocated services such as the audio and video service. After the app received the unique resource locators (URLs) of services, it requested the services so that channels between the app and server were built up. (2) The agent sent instructions to the user. The server played a role as a broker. The lighting region in the diagram means that all channels had been built. (3) The user terminated the app. The three components worked collaboratively with a clear temporal design, and their sub-modules were decoupled, so the system’s robustness was enhanced, making it safer and more reliable.

To continually earn the trust of a wide user base, the software of this system must support concurrent usage by multiple users. Moreover, to fully leverage the interactive capabilities between physical devices and users, the software also needs to be scalable in functionality and maintainable in structure. Following the iterations of hardware design based on feedback from visually impaired users, the software components, including the mobile app, server, and frontend, have been refined into the following modular design.

#### 2.2.1. Mobile Phone App

The role of the mobile app is to serve as a data transmission hub. During the process of assisting visually impaired people, it serves as the interface that allows users to log in to the system and make travel reservations through voice commands. Once the travel service is launched, it is responsible for preprocessing and integrating various sensor data before uploading them to the server side, such as multi-channel video, audio, and IMU (Inertial Measurement Unit) data. The mobile app also needs to receive instructions from the server, decode them, and transmit them to the controller in the helmet.

**Figure 4:**
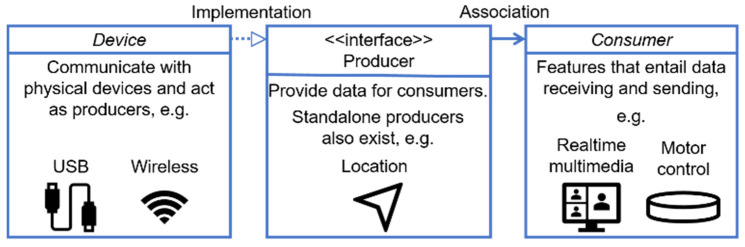
Diagram of the key classes of the mobile app. The key concepts included the device, producer, and consumer. A device class communicated with a physical device. A class implementing producer interface either provided data for or requested feedback from consumers. A consumer stood for a feature that involves data transmission.

By abstracting hardware devices and services, we adopted Object-Oriented Programming (OOP). This approach endowed the system with excellent scalability and maintainability. Figure 4 illustrates some of the key classes in the object-oriented design. The device represents an abstraction of various embedded systems. Devices could connect to the mobile phone either with a cable or wirelessly, acting as producers to provide data for consumers. A producer role could also be played by the phone itself; in addition to producing sensor data, some producers needed to receive feedback from consumers. Consumers acted as data intermediaries with input capabilities. They obtained and processed data from the network or producers, and then fed it back to other producers, enabling complex data transmission and information distribution. Such a design decoupled services from hardware devices and allowed visually impaired people to utilize the previously mentioned helmet and even more advanced future devices to receive the same type of services, without needing to modify the codes related to services themselves.

#### 2.2.2. Server

As a bridge between mobile app and backend agent, the server’s role is to provide an Internet-accessible entry point for them, ensuring that various channels can be correctly established for each service request. To ensure scalability of the system, the server is composed of multiple independent microservices, including the authentication service, the data exchange service for navigation, and a third-party audio and video service. The authentication service allocates entries to the data exchange and audio–video services for the mobile app and agent frontend. This allows the system’s scale to dynamically adjust according to demand. The independence of each microservice also enhances the system’s reliability.

#### 2.2.3. Backend Agent

In addition to widely used artificial intelligence (AI) recognition technology, the backend is a key component designed for the wearable blind assistance system, integrating real-time video streams from dual cameras, map location and operation instruction display, as well as input/output functions like tactile and voice feedback. The technical implementation of the backend interface considers usability and efficiency. By utilizing modern web technologies such as WebSocket for real-time data transmission and Protobuf for data serialization, the system ensures smooth data interaction and a rapid system response. Moreover, the design of the system also takes into account scalability, to potentially integrate more functions and services in the future.

**Figure 5:**
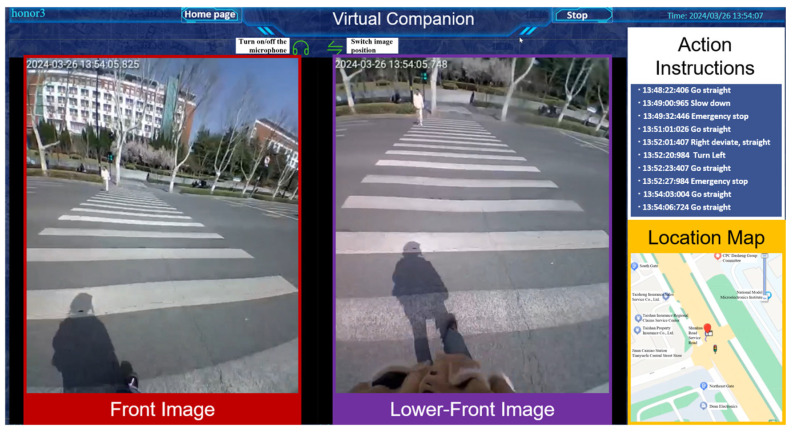
Diagram of backend interface functions. There are four main functional parts: the two image boxes on the left show the user’s front and lower-front environment transmitted back from the helmet cameras. The upper right functional area displays the action instructions sent by the operator to the user in chronological order, and the lower right displays the user’s current location.

It aims to provide visually impaired users with real-time remote companionship and obstacle avoidance navigation, enhancing their sense of security and trust. The operator observes the user’s surrounding environment in real time through the backend interface (Figure 5), and provide real-time guidance and interaction to users through tactile and voice feedback, including the acquirement of user requirements, map navigation, and path planning.

### 2.3. Instruction Set

The main ways for backend operators to guide blind helmet wearers are tactile encoding and voice feedback. Tactile coding feedback refers to the backend operator guiding the wearer to walk by manipulating the joystick controller (model: PXN-2113; manufacturer: PXN, Shenzhen, China). The instructions issued by the joystick controller in the background are decoded by the mobile app, causing motor vibration inside the helmet, which is transmitted to the wearer through tactile means, and supplemented by automatic voice broadcasting of relevant action instructions. The instruction set of tactile feedback, which includes the agreed relationship between instruction purpose, handle operation, motor vibration encoding, and automatic voice broadcasting, has evolved to a well-accepted version after undergoing continuous optimization [16], as shown in Table 1. Considering the convenience of backend and user learning and applications, as well as the need for safe travel, this instruction set has made two improvements. One is the decrease in the number of motors and their vibration combinations that trigger tactile sensation, which helps users become familiar with the system faster. And the other is the use of joystick controllers (PXN-2113) instead of keyboards for backend personnel to issue action commands, which reduces the feedback latency of the system.

### 2.4. Methods of System Usage

Users can make appointments via phone or by logging into the app in accessibility mode. During use, the system automatically starts and connects to the backend once the helmet’s Type-C port is plugged into an Android phone. On first use, the system guides the user through a one-click authorization process in accessibility mode to grant necessary permissions such as location access and voice input/output. This authorization is persistent and does not need to be repeated in future uses. For non-first-time use, the device automatically connects to the cloud backend and synchronizes the user’s identity information upon connection. During operation, the backend agent displays the user’s real-time location and environmental status. Backend operator initiates tactile and auditory interaction with the wearer through a joystick controller and microphone. The system can be used independently without assistance from others.

### 2.5. Methods of System Testing

The system testing method adopted in this study is based on the theoretical framework of the extensional definition of tests [17]. To ensure the consistency and independence of the results, each testing scenario was assigned a specific combination of tactile and auditory feedback. Additionally, the system was re-initialized prior to each test.

Subjects in this project include two groups of people: college students with normal vision or corrected vision, and visually impaired individuals (application No. ECSBMSSDU2024-1-94). All volunteers had no prior experience with the MNVCS.

Prior to formal testing, all subjects were trained to use the system in a room outside of a testing scenario for about 5 min. Subjects were not informed of the testing path until all testing had been completed, and were blindfolded when entering and exiting the testing site.

The testing for MNVCS is divided into three stages.

Stage 1: Test for Easy-to-Learn Performance. For safety reasons, we initially recruited 12 non-blind graduate students (6 females and 6 males; aged 23–26) from Shandong University. They wore eye masks during testing.

Stage 2: Test for Transferability of the Usage Experience. To test whether the MNVCS can truly assist the blind in their travel, which is the purpose of this technology, visually impaired individuals with no or little visual or light sensitivity were recruited in Zhuzhou, Hunan Province, with a total of 18 participants (8 females and 10 males; 5 with a primary school degree, 3 with a junior high school degree, 3 with a high school degree, 4 with a technical secondary school education, and 3 with a college degree). Among them, individuals with different levels of visual impairment were distributed as follows: Level 1 (severe blindness)—16 individuals; Level 2 (mild blindness)—2 individuals. The age range was from 32 to 67 years, with an average age of 47.7 years. None of the participants carried a cane during the test.

Stage 3: Test for Safety and Reliability in an Outdoor Scene. Six non-blind college student subjects (six females: one undergraduate student and five graduate students, aged 22–25) from Shandong University and eight visually impaired individuals with no visual or light sensitivity (eight males: two with a junior high school degree, two with a high school degree, and four with a technical secondary school education) were recruited in Zhuzhou, Hunan Province. Considering the familiarity demands of visually impaired individuals in real-life scenarios, the blind participants were given the option to use their canes while being equipped with our system. The outdoor testing in this phase was conducted on clear days during the daytime. The testing sites included open street blocks and park roads. The environments were unfamiliar to the visually impaired participants, aiming to simulate the challenges of navigating in unfamiliar but real-world settings.

The use of canes followed the daily habits of visually impaired subjects without any special requirements. The system did not interfere with the natural use of the cane. To ensure the safety of participants throughout the testing process, a dedicated on-site safety supervisor was assigned for each test session. During testing, supervisors were not allowed to proactively intervene or safeguard the participant through verbal or physical means, unless the participant’s safety was at risk. The system allowed participants to independently terminate the testing process at any time based on their personal condition. If no interactive input was received for 30 consecutive seconds or communication is lost, the wearable system would automatically treat the test as terminated and issue a voice alert to notify the participant. Prior to testing, all participants received comprehensive safety training and signed a written informed consent form, confirming their full understanding and acceptance of the emergency safety protocols and contingency procedures.

## 3. Results

### 3.1. Easy-to-Learn Performance

**Figure 6:**
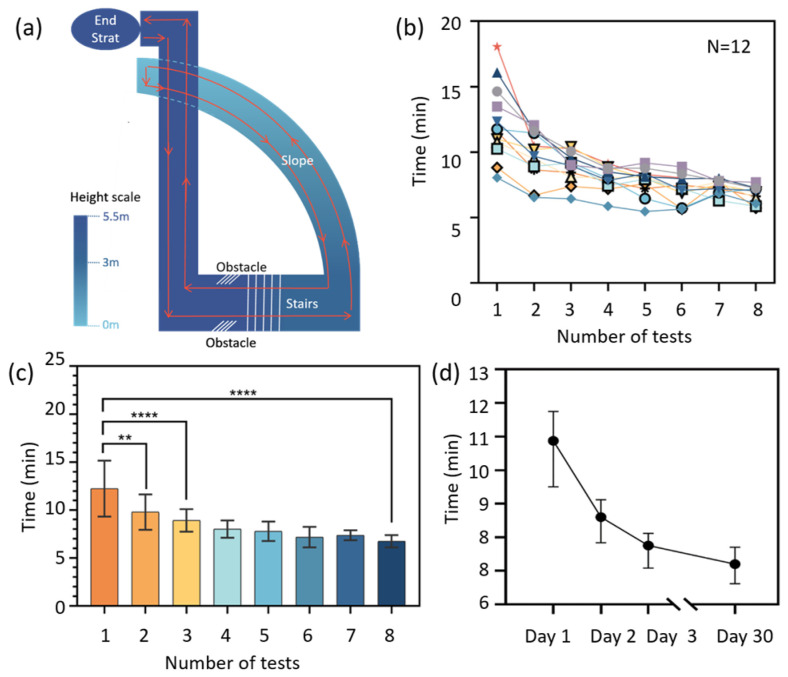
The easy-to-learn performance of the navigation system. (**a**) A diagram of the testing route. (**b**) The decrease in walking time with the usage time. (**c**) The relationship between user proficiency and testing number. Each column represents the average walking time, with error bars indicating standard deviation. The significant differences are as follows: ** *p* ≤ 0.01, **** *p* < 0.0001. Data in (**d**) show the median testing time of all 12 subjects.

The study tested the relationship between the time required to walk on a fixed route while wearing this device and recorded the number of uses, the time taken to complete the route, and whether or not the participant used a blind cane as an aid. The selected test route, with a total length of about 500 m, was a closed loop in the Software Park Campus of Shandong University, including straight sections, turns, and stairs, as shown in Figure 6a. After a 5-min training session in which they learned how to use the device, the participants underwent two stages of fixed route testing: (1) with the assistance of a cane, completing two tests per day for three consecutive days, and (2) 30 days later, they completed four tests, two using a blind cane and two without the use of a blind cane. One subject declined to participate in the test 30 days later due to leaving the school. All the testing data are listed in Table S1 in Supplementary Material.

Figure 6b shows that the walking time of 12 subjects on the fixed route decreased as the usage count increased, and then reached a relatively steady level. In the first test, due to individual differences in learning ability, courage, and trust in the product, the walking time varied significantly, ranging within 8–18 min. By the eighth test, which was conducted one month later, the walking time of all participants had decreased to around 8 min, with a smaller range of variation from 7 to 9 min. This demonstrates that the experience, trust and proficiency of participants in using the product all increase as their usage of the device increases.

Figure 6c,d show a significant decrease in walking time between Test 1 and Test 2 and between Day 1 and Day 2 (in Figure 6d), further demonstrating the ease of use of this device, which means that it can be proficiently applied without requiring extensive training and practice.

Thirty training-free days later, the walking time of the volunteers still decreased slightly, as shown in Figure 6d. This indicates that, though without continuous training or use, the obtained experience, trust and proficiency do not disappear over time for a long period like one month, demonstrating the operability and learnability of the device.

### 3.2. Transferability of Usage Experience

**Figure 7:**
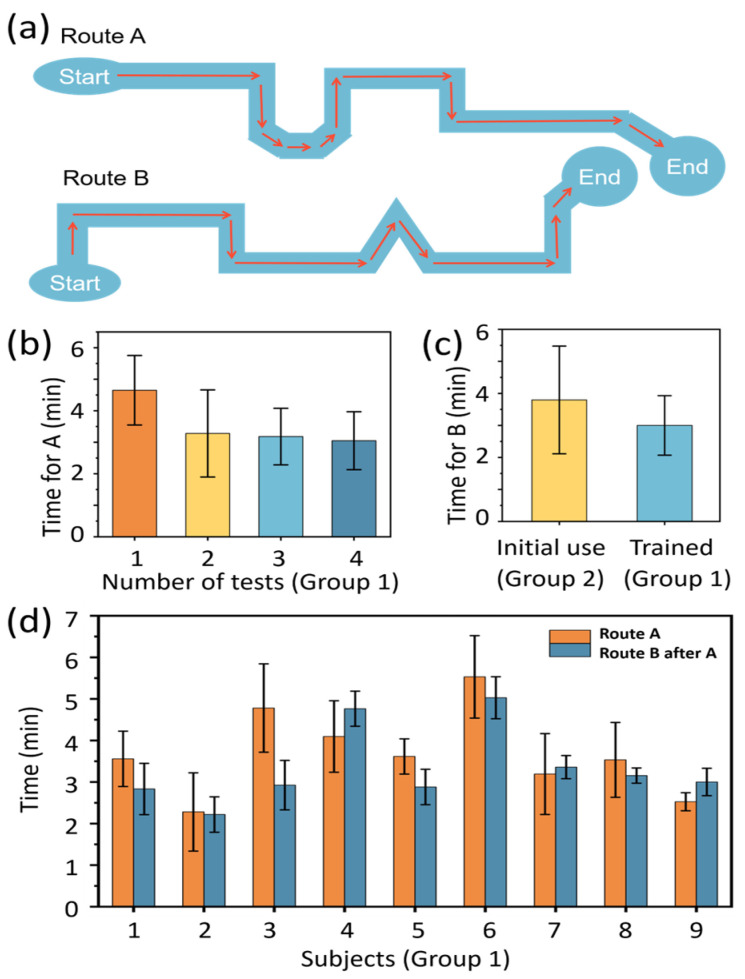
Transferability of usage experience of the navigation system. (**a**) Schematic diagram of the test routes. (**b**) Walking time of Group 1 subjects on Route A. (**c**) Walking time of Group 1 and Group 2 subjects on Route B. (**d**) Comparison of walking time on Route A and Route B for Group 1 subjects. Each column represents the average walking time on the same route, with error bars indicating standard deviation.

We tested the relationship between the time required to walk on a fixed route while wearing this device and whether one has been trained. Two fixed routes, A and B, with lengths of approximately 60 m each, were chosen for testing. These routes were located indoors at the Zhuzhou Disabled Persons’ Federation and both included elements such as straight sections, turns, and entering/exiting the room, as shown in Figure 7a. After an approximately 5 min training session on device usage, the blind subjects were divided into two groups. The first group (Group 1, nine subjects) and another two non-blind subjects walked route A four times in one day, followed by walking route B four times the next day. The second group (Group 2, nine subjects) walked route B four times in one day, without prior training on route A. All testing data are listed in Table S2 in the Supplementary Material.

Similar to the test results of student subjects, both for route A and route B, the blind subjects’ walking time decreased with an increase in usage count during the initial test, and eventually reached a relatively stable level, as shown in Figure 7b for route A and Figure S1 for route B in the Supplementary Material.

Figure 7c,d show the walking time on route A and that on the following route B of every subject in Group 1. Two-thirds of the subjects who received training on route A had a significantly shorter walking time on route B. However, it is worth noting that subjects 4, 7, and 9 took more time to complete Route B during the second walk. This individual variation is considered to be primarily related to age-associated sensitivity changes in tactile and auditory input (e.g., subject 4 was 51 years old; subject 7 was 54 years old), or impaired motor function (e.g., subject 9), which affected their responsiveness to the system’s instructions. On the other hand, subject 3 completed the task in significantly less time than the average, possibly owing to his self-reported richer travel experience, higher acceptance of new devices, and more decisive operation. Despite the above individual differences in performance, combined with the comparison of the walking time taken for walking route B for the first time (initial use, 4 min) and walking route B after training on route A (trained, 3 min) in Figure 7c, it can be found that the usage experience of the developed navigation device is transferable. Furthermore, this also indicates that with an increase in usage count, the participants increased their trust in using the product, and became more proficient in its usage. Moreover, this improvement in trust, proficiency, and experience does not depend on user’s familiarity with the specific fixed routes walked, but rather on the transferability of the acquired usage experience.

### 3.3. Safety and Reliability in Outdoor Scene

**Figure 8:**
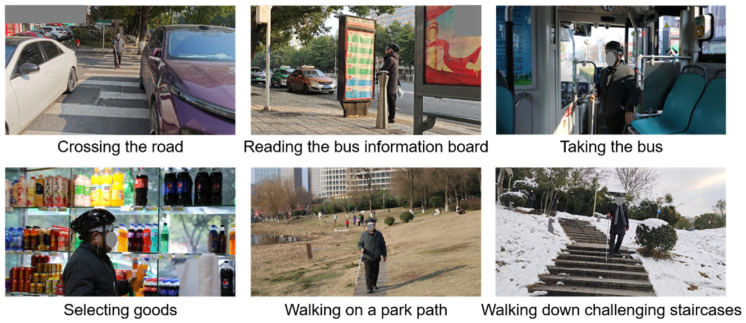
Tests of the system in real-life scenarios, including but not limited to crossing the road, reading the bus information board and taking the bus, going shopping to select goods, walking on a park path, and walking up and down consecutive staircases.

With the help of the MNVCS, the blind participants were not only able to walk safely in both open areas and narrow passages, but also to fulfil challenging tasks under the navigation and/or guidance of the system, such as crossing the road, reading the bus information board and taking the bus, going shopping to select goods, walking in a park, and walking up and down consecutive stairs, as illustrated in Figure 8. Scene testing videos for cycling, transportation and stair descending can be found in Video S1 in the Supplementary Material.

## 4. Discussion

### 4.1. Satisfaction Survey

As mentioned in the Introduction, the types of interaction and the wearer’s trust are crucial for the promotion of assistive electronic devices. All subjects, including both the non-blind and the blind volunteers, appeared to become familiar with the MNVCS’s human–machine interaction protocol after only 5 min of training. The above Results proved the practicality of multimodal feedback, which consists of 90% tactile feedback for navigation and 10% auditory feedback for urgent reminders and necessary personalized communication with backend agents. According to the survey questionnaire, 90% of visually impaired participants expressed that when using virtual companionship services, they feel more psychologically secure and have the courage to engage in outdoor activities or travel alone.

Therefore, the following features were proven to contribute to enhancing the practicality and widespread acceptance of visual substitution products.

Safety and reliability are the primary requirements for visual substitution devices. Here, remote navigation and virtual companionship are the most crucial type of support for the self-developed system’s future expansion to serve vulnerable groups such as the elderly and children.

Wearable, hands-free operation: wearables can free up the wearer’s hands and provide flexibility.

Liberate hearing: external information feedback should minimally (or preferably not at all) affect the user’s auditory senses, since the hearing is an important sense that allows blind users to perceive sudden external situations. Here, tactile feedback is adopted as the main feedback approach, with auditory feedback as a supplement.

Simplicity and ease of use: it is recommended to avoid including unnecessary features in a device’s operation and interface, ideally enabling one-touch activation without the need for extensive training periods. The five-minute short-term training session and the transferability of usage experience described in our study have both proven the practicality of this MNVCS.

Table 2 lists the key functions and limitations of several blind assistance devices currently available on the market and the MNVCS we have developed here.

**Table 2:** Analysis of existing blind travel assistance devices.

Product	Function	Limitations
OrCam MyEye [18] Be My Eyes [19]	The glass describes text, faces, product, colors, and so on, to the blind person vocally. The range of vision is 1.5–9 m.	Auditory occupancy. No navigation and obstacle avoidance. Dependent on lighting conditions.
Aira [20]	The produce is hand-held. The backend agent informs the blind person of the surrounding environment through voice.	Auditory occupancy. Hand occupancy. Requires monthly subscription.
NavCog [21] Blind Square [22]	The applications use the phone’s GPS, combined with both voice and phone vibration, to help the blind person navigate.	Hand occupancy. No obstacle avoidance reminder. Location information is updated slowly.
Sunu Band [23]	This watch is equipped with a sonar sensor, with a visual range of over 6.8 m. It navigates and reminds users to avoid obstacles by vibrating the wristband or emitting a beep sound.	Fixed usage gestures. High price.
Smart Cane [24]	The device is hand-held. It can detect obstacles within 3 m via ultrasound, and can help the blind person to navigate and identify obstacles through different vibration modes and sounds.	The detection range is insufficient to handle complex environments. Not in line with the habit of blind people using canes.
MNVCS (This work)	The wearable system provides multimodal navigation, obstacle avoidance, and virtual companion services, featured with hands-free, less use of hearing, and a stronger sense of security.	Awaiting lightweighting. Not yet industrialized.

### 4.2. Latency Effects

The overall system latency for the closed-loop operation is approximately 1.0–1.5 s. This is primarily attributed to the higher frame rate of image acquisition by blind individuals wearing cameras (approximately 20–30 frames per second) and the inevitable delays in information processing and communication. Even so, the latency has minimal impact on the overall system usage. On one hand, the walking speed of blind individuals is slow, ranging from 0.5 to 1.0 m per second. On the other hand, experienced backend personnel can preemptively issue action instructions to compensate for the latency.

At present, the acquired image information from cameras worn by blind individuals exhibits minimal differences between adjacent frames, with information redundancy exceeding 90%. The wireless transmission of high-frame-rate camera images consumes significant communication bandwidth, necessitating compression. In practical scenarios, such as walking in open scenes, board games, shelf selection, and other routine activities, frame rate control of 3–5 frames per second for continuous imaging is sufficient to provide a substantial basis for backend personnel to make quick and accurate judgments. Reducing redundant information transmission between the frontend and backend is a technical optimization point that can decrease the overall system latency. There is a prospect of reducing the total delay from the current 1.0–1.5 s to 0.1–0.3 s.

### 4.3. Difference in Non-Blind and Blind Subjects

It is worth noting that some aspects of the non-blind subjects’ testing experience is not entirely applicable to patients with visual impairments.

**Figure 9:**
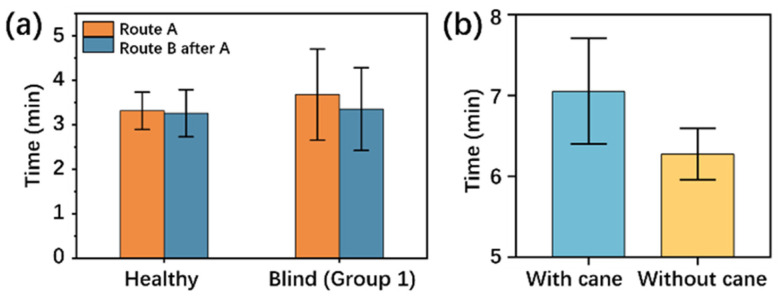
Difference testing results in non-blind and blind subjects. (**a**) Walking time comparison between non-blind and blind subjects on Route A and Route B. (**b**) Walking time comparison with and without the use of a blind cane on a fixed route. The column height is the average walking time on the same route, and the error bar is the standard deviation.

Figure 9a shows the differences in results between non-blind and blind subjects in the same test, i.e., completing route A followed by route B. Compared with walking time on route A, there is no obvious decrease in walking time on route B among non-blind subjects, while the difference in the walking time for routes A and B is relatively obvious.

Figure 9b shows the difference in walking time of the subjects when they walked with the navigation device on the fixed route with and without the use of a blind cane. Subjects’ walking time decreased from 8 min when using the cane to around 7 min when the cane was not used, seeming to indicate that the assistance of a cane is an optional rather than a mandatory option for the wearer. However, this should be because non-blind individuals possess more intuitive judgment of their external environment, so using a blind cane—for example, by sweeping it on the road—can slow down a person’s walking speed. This phenomenon does not apply to patients with visual impairments.

The above two cases indicate that tests on visual impairments are more valuable, even though the easy-to-learn performance of non-blind student subjects was replicated in the tests involving visually impaired subjects, as shown in Figure 7b and Figure S3 in the Supplementary Material.

### 4.4. Overview

In the future, we will undertake the following technical optimizations and application expansions for the remote blind navigation system:

(1) We will develop a reasonable camera image compression algorithm to reduce redundant image transmission occupying communication traffic. According to the users’ needs, the continuous shooting frame rate is controlled at 3–5 frames/second, and the background algorithm is used to “fill” the slowly changing images between adjacent frames, forming a background visual effect of “continuous shooting” which is “like being on the scene”, which helps the background personnel make quick and accurate judgments. This also helps reduce the latency of the entire system’s frontend and backend closed-loop operations from the current 1.0–1.5 s to 0.1–0.3 s.

(2) We will provide enhanced and more complete 3D map twins of pedestrian spaces and indoor space scenes and real-time dynamic “bird’s-eye” perspective information, and improve the wearer’s spatial positioning accuracy to about 0.1 m.

(3) Based on the premise of retaining the full participation of backend personnel in navigation and roles, we will gradually enhance edge computing capabilities, increase the proportion of wearable systems making on-site AI judgments and decisions, and gradually reduce the attention and decision-making content required from backend personnel. In doing so, we hope to achieve more efficient backend personnel service in a “one-to-many” scenario.

(4) Introducing individuals with unimpaired vision but who have other physical disabilities as backend personnel helps create inclusive employment opportunities for disabled people. Meanwhile, the system’s application boundaries can be further extended through the development of derivative products tailored to specific scenarios. For example, a heat-resistant helmet system for firefighters could provide remote expert guidance in complex fire scenes; lightweight daily assistance devices could accompany and facilitate the daily lives of elderly individuals living alone or in care institutions; and real-time remote-support equipment could serve expeditions and scientific research in extreme outdoor environments. These derivative products will retain the core architecture of “remote real-time navigation combined with multimodal frontend sensing and feedback”, with hardware and functionalities customized and optimized for each application scenario.

## 5. Conclusions

Compared to other devices or systems which also utilize network apps and involve volunteers in AI-assisted recognition, the system proposed in this article fully considers the subjective initiative of visually impaired individuals. Through the comprehensive integration of software, hardware, and human involvement, and joint participation of the user and backend personnel, this approach provides continuous navigation and action instructions, offering both physical and psychological security to the user. The core concept of remote companionship can give the user a sufficient sense of security and provide personalized services. The integration of multimodal feedback systems enhances the stress responses of the system in various scenarios, thus improving the overall physical safety factor. By using tactile codes as feedback orders, the current system frees the user’s hands and keeps its interference with hearing function to a minimum, preserving the user’s perception and response to external emergencies. However, it also offers a direct sense of orientation. This can reduce the user’s reaction time, unlocking more independent living scenarios. In the future, this system can also be promoted for use among other vulnerable groups such as the elderly. With additional support and further developments such as techniques for creating precise and dynamic 3D maps of the surrounding environment, positioning the location of the user to 0.1 m, as well as on-site AI-assisted image recognition, the present navigation system will have a bright, promising future.

## Figures and Tables

**Table 1:** The instruction set of tactile feedback.

Purpose of Command	Joystick Operation	Vibration Coding	Automatic Voice	Notes
Preparation	Press the prepare button.	Front, rear, left and right vibrate clockwise for 0.2 s, with an interval of 0.2 s between each vibration.	Ready to go.	Initial preparation: test motors and observe environment.
(Keep) going straight	Push the handle to the front position every 2 s.	The front motor vibrates for 0.2 s.	/	Start or keep going straight.
Slow down	Push the handle to the rear position.	The rear motor vibrates twice continuously for 0.2 s each at 0.2 s intervals.	Slow down.	Slow down, wait and see to ensure safety.
Emergency stop	Press the stop button.	Front, rear, left and right motors vibrate simultaneously for 0.3 s.	Stop!	Avoid danger and observe road conditions.
Deviate to the left and go straight	Push the handle to the left position.	The front motor vibrates for 0.2 s, and 0.2 s later, the left motor vibrates for 0.1 s.	/	Bypass the obstacle.
Deviate to the right and go straight	Push the handle to the right position.	The front motor vibrates for 0.2 s, and 0.2 s later, the right motor vibrates for 0.1 s.	/	Bypass the obstacle.
Turn left	Press the left turn button.	The left motor vibrates three times continuously for 0.1 s each at 0.2 s intervals.	Turn left.	Turn left.
Turn right	Press the right turn button.	The right motor vibrates three times continuously for 0.1 s each at 0.2 s intervals.	Turn right.	Turn right.
Prepare to go up/down the steps	Voice prompt: “About two steps ahead to the stairs, the handrail is on your left/right hand, go up/down the stairs carefully”.			Instruct the blind person to feel the position of the steps and handrails with hands, feet or blind sticks.
Up/down the steps	Voice prompt: “This is the first step” and/or “This is the last step”.			Instruct the blind person to start going up/down the steps.

## Data Availability

The original contributions presented in this study are included in the article/Supplementary Material. Further inquiries can be directed to the corresponding author(s).

## Supplementary Materials

The following supporting information can be downloaded at https://www.mdpi.com/article/10.3390/s25134223/s1, Figure S1: The downward trend of walking time of the blind subjects (Group 2) with the increasing usage count on Route B; Table S1: Testing data of twelve student subjects; Table S2: Testing data of subjects with visual impairments; Video S1: Scene testing videos of cycling, transportation, and stair descending.
