# Supplementary material for: Multimodal Navigation and Virtual Companion System: A Wearable Device Assisting Blind People in Independent Travel
Source: Sensors (Basel). 2025 Jul 6;25(13):4223. doi: 10.3390/s25134223 (PMC12252471; doi:10.3390/s25134223)
Supplement: Supplementary file 1 [file sensors-25-04223-s001.zip › Supplementary materials.pdf]

Multimodal Navigation and Virtual Companion System: A Wearable Device Assisting Blind People in Independent Travel

Jingjing Xu, Caiyi Wang, Yancheng Li, Xuantuo Huang, Meina Zhao, Zhuoqun Shen, Yiding Liu, YuXin Wan, Fengrong Sun, Jianhua Zhang and Shengyong Xu

Table S1. Testing data of twelve student subjects.

Subject number	Date	Usage count	Duration	Walking time (min)
1	Day 1	1	10:46:22 - 11:01:00	14.63
		2	11:28:18 - 11:39:54	11.60
	Day 2	3	11:02:22 - 11:12:26	10.07
		4	11:27:45 - 11:36:31	8.77
	Day 3	5	10:55:35 - 11:04:21	8.77
		6	11:36:22 - 11:44:42	8.33
	Day 30	7	14:16:23 - 14:24:12	7.82
		8	14:50:42 - 14:57:56	7.23
		without blind cane	15:33:34 - 15:39:51	6.28
		without blind cane	16:02:37 - 16:08:56	6.32
2	Day 1	1	11:08:35 - 11:22:04	13.48
		2	11:45:20 - 11:57:23	12.05
	Day 2	3	10:47:00 - 10:56:00	9.00
		4	11:17:18 - 11:26:01	8.72
	Day 3	5	14:26:53 - 14:36:03	9.17
		6	14:39:24 - 14:48:17	8.88
	Day 30	7	14:27:31 - 14:35:18	7.78
		8	15:00:33 - 15:08:15	7.70
		without blind cane	15:42:58 - 15:49:08	6.17
		without blind cane	16:11:16 - 16:17:24	6.13
3	Day 1	1	15:14:43 - 15:30:48	16.08
		2	16:08:58 - 16:20:37	11.65
	Day 2	3	10:22:44 - 10:32:17	9.55
		4	11:16:14 - 11:24:43	8.48
	Day 3	5	10:27:10 - 10:35:17	8.12
		6	11:35:20 - 11:43:18	7.97
	Day 30	7	14:40:21 - 14:48:21	8.00
		8	15:12:09 - 15:19:25	7.27
		without blind cane	15:24:11 - 15:30:53	6.70
		without blind cane	15:52:30 - 15:59:20	6.83
4*	Day 1	1	14:47:01 - 15:05:04	18.05
		2	15:44:33 - 15:54:50	10.45

	Day 2	3	14:53:59 - 15:04:15	10.27
		4	15:06:23 - 15:15:30	9.12
	Day 3	5	11:53:45 - 11:02:02	8.28
		6	11:04:08 - 11:12:10	8.03
5	Day 1	1	14:23:41 - 14:36:01	12.33
		2	14:49:06 - 14:58:47	9.68
	Day 2	3	14:17:01 - 14:25:58	8.95
		4	14:37:03 - 14:44:53	7.83
	Day 3	5	14:15:39 - 14:24:00	8.35
		6	14:34:30 - 14:41:35	7.08
	Day 30	7	14:19:55 - 14:27:07	7.20
		8	14:50:56 - 14:57:59	7.05
		without blind cane	15:08:18 - 15:14:39	6.35
		without blind cane	15:23:29 - 15:30:08	6.65
6	Day 1	1	14:05:19 - 14:13:21	8.05
		2	14:38:47 - 14:45:19	6.53
	Day 2	3	14:06:50 - 14:13:16	6.43
		4	14:28:46 - 14:34:38	5.87
	Day 3	5	14:03:08 - 14:08:36	5.47
		6	14:25:59 - 14:31:37	5.63
	Day 30	7	14:09:55 - 14:16:46	6.85
		8	14:42:51 - 14:48:55	6.07
		without blind cane	15:00:12 - 15:06:10	5.97
		without blind cane	15:16:31 - 15:22:29	5.97
7	Day 1	1	13:48:09 - 13:59:54	11.75
		2	14:19:55 - 14:31:22	11.45
	Day 2	3	14:03:46 - 14:12:58	9.20
		4	14:30:22 - 14:38:21	7.98
	Day 3	5	14:08:30 - 14:14:57	6.45
		6	14:30:29 - 14:36:12	5.72
	Day 30	7	15:06:45 - 15:13:39	6.90
		8	15:18:53 - 15:26:05	7.20
		without blind cane	15:27:53 - 15:34:20	6.47
		without blind cane	15:35:26 - 15:41:31	6.08
8	Day 1	1	14:06:15 - 14:16:30	10.25
		2	14:46:15 - 14:55:09	8.90
	Day 2	3	14:17:08 - 14:26:19	9.18
		4	14:41:03 - 14:48:29	7.43
	Day 3	5	14:17:49 - 14:25:44	7.92
		6	14:39:27 - 14:46:40	7.22
	Day 30	7	10:37:37 - 10:43:55	6.30
		8	10:44:52 - 10:50:44	5.87
		without blind cane	10:52:12 - 10:58:27	6.25
		without blind cane	10:59:00 - 11:04:45	5.75
9	Day 1	1	15:05:10 - 15:15:55	10.75
		2	15:37:32 - 15:47:02	9.50

	Day 2	3	15:02:50 - 15:10:56	8.10
		4	15:26:02 - 15:34:05	8.05
	Day 3	5	14:59:42 - 15:07:47	8.08
		6	15:22:36 - 15:29:47	7.18
	Day 30	7	10:03:56 - 10:11:38	7.70
		8	10:45:16 - 10:51:44	6.47
		without blind cane	10:26:52 - 10:33:16	6.40
		without blind cane	10:34:56 - 10:40:47	5.85
10	Day 1	1	15:20:16 - 15:31:16	11.00
		2	16:02:46 - 16:12:58	10.20
	Day 2	3	15:13:20 - 15:23:44	10.40
		4	15:36:51 - 15:45:42	8.85
	Day 3	5	15:10:05 - 15:18:04	7.98
		6	15:40:39 - 15:47:32	6.88
	Day 30	7	16:32:00 - 16:39:31	7.52
		8	16:42:06 - 16:49:03	6.95
		without blind cane	16:50:03 - 16:56:24	6.35
		without blind cane	16:56:55 - 17:03:33	6.63
11	Day 1	1	16:29:45 - 16:38:34	8.82
		2	16:56:40 - 17:03:22	6.70
	Day 2	3	16:07:56 - 16:15:19	7.38
		4	16:29:23 - 16:36:34	7.18
	Day 3	5	16:02:31 - 16:10:06	7.58
		6	16:21:52 - 16:27:31	5.65
	Day 30	7	15:59:38 - 16:07:07	7.75
		8	16:09:09 - 16:14:53	5.73
		without blind cane	16:15:56 - 16:22:09	6.22
		without blind cane	16:23:39 - 16:29:16	5.62
12	Day 1	1	16:41:49 - 16:53:23	11.57
		2	17:05:47 - 17:14:25	8.63
	Day 2	3	16:18:22 - 16:26:48	8.43
		4	16:38:57 - 16:46:42	7.75
	Day 3	5	16:12:16 - 16:19:29	7.22
		6	16:30:09 - 16:37:38	7.48
	Day 30	7	10:03:20 - 10:10:32	7.20
		8	10:12:00 - 10:18:37	6.62
		without blind cane	10:19:47 - 10:26:19	6.53
		without blind cane	10:27:20 - 10:33:52	6.53

* The subject did not participate in the test 30 days later due to leaving the school.

Table S2. Testing data of subjects with visual impairments.

S2-1. Test data of subjects in Group 1				
**Subject number**	**Route**	**Usage count**	**Duration**	**Walking time (min)**
1	A	1	10:15:26 - 10:19:58	4.53
		2	10:30:46 - 10:33:59	3.22
		3	10:48:49 - 10:52:15	3.43
		4	11:09:16 - 11:12:19	3.05
	B	1	9:53:31 - 9:57:08	3.62
		2	10:00:11 - 10:02:31	2.33
		3	10:22:25 - 10:25:27	3.03
		4	10:26:56 - 10:29:17	2.35
2	A	1	16:17:22 - 16:21:02	3.67
		2	16:24:39 - 16:26:34	1.92
		3	16:34:34 - 16:36:31	1.95
		4	16:38:20 - 16:39:55	1.58
	B	1	13:37:29 - 13:40:18	2.82
		2	13:41:38 - 13:43:48	2.17
		3	13:54:09 - 13:56:12	2.05
		4	13:57:57 - 13:59:47	1.83
3	A	1	10:21:44 - 10:27:44	6.00
		2	10:29:55 - 10:35:15	5.33
		3	10:37:25 - 10:41:09	3.77
		4	10:43:37 - 10:47:38	4.02
	B	1	13:06:44 - 13:10:06	3.37
		2	13:17:27 - 13:20:57	3.50
		3	13:23:55 - 13:26:25	2.50
		4	13:28:20 - 13:31:03	2.33
4	A	1	11:10:19 - 11:15:33	5.23
		2	11:23:55 - 11:27:13	3.30
		3	11:51:58 - 11:56:14	4.27
		4	11:58:30 - 12:02:05	3.58
	B	1	13:50:42 - 13:55:47	5.08
		2	13:58:09 - 14:02:58	4.82
		3	14:05:19 - 14:10:19	5.00
		4	14:27:50 - 14:31:59	4.15
5	A	1	14:48:29 - 14:52:33	4.07
		2	14:54:06 - 14:57:58	3.87
		3	15:00:14 - 15:03:24	3.17
		4	15:06:05 - 15:09:26	3.35
	B	1	16:24:06 - 16:27:35	3.48
		2	16:29:37 - 16:32:09	2.53
		3	16:34:03 - 16:36:56	2.88
		4	16:38:10 - 16:40:48	2.63
6	A	1	15:26:27 - 15:32:56	6.48
		2	15:58:33 - 16:04:50	6.28

		3	16:58:31 - 17:03:19	4.83
		4	17:05:09 - 17:09:41	4.53
	B	1	9:46:31 - 9:52:00	5.48
		2	9:53:49 - 9:59:15	5.43
		3	10:19:28 - 10:24:12	4.73
		4	10:25:52 - 10:30:20	4.47
7	A	1	11:11:10 - 11:15:49	4.65
		2	11:18:00 - 11:20:38	2.63
		3	11:22:58 - 11:25:44	2.77
		4	11:29:51 - 11:32:34	2.72
	B	1	13:39:04 - 13:42:39	3.58
		2	13:45:47 - 13:49:22	3.58
		3	13:51:08 - 13:54:08	3.00
		4	13:56:34 - 13:59:50	3.27
8	A	1	14:21:49 - 14:26:40	4.85
		2	14:29:13 - 14:32:30	3.28
		3	14:33:57 - 14:37:08	3.18
		4	14:40:10 - 14:42:59	2.82
	B	1	16:03:10 - 16:06:35	3.42
		2	16:08:06 - 16:11:11	3.08
		3	16:13:44 - 16:16:44	3.00
		4	16:18:22 - 16:21:29	3.12
9	A	1	10:13:01 - 10:15:47	2.77
		2	10:16:59 - 10:19:37	2.63
		3	10:21:08 - 10:23:23	2.42
		4	10:24:43 - 10:27:00	2.28
	B	1	12:57:31 - 13:00:58	3.45
		2	13:02:10 - 13:04:52	2.70
		3	13:06:06 - 13:08:55	2.82
		4	13:13:01 - 13:16:03	3.03

S2-2. Test data of subjects in Group 2

Subject number	Route	Usage count	Duration	Walking time (min)
1	B	1	14:22:11 - 14:26:17	4.10
		2	14:30:37 - 14:34:55	4.30
		3	14:49:23 - 14:55:00	5.62
		4	14:56:24 - 15:00:15	3.85
2	B	1	15:12:03 - 15:16:42	4.65
		2	15:19:26 - 15:22:31	3.08
		3	15:30:55 - 15:34:47	3.87
		4	15:37:04 - 15:39:18	2.23
3	B	1	9:56:44 - 10:09:03	12.32
		2	10:13:18 - 10:21:27	8.15
		3	10:35:27 - 10:43:16	7.82
		4	10:47:49 - 10:53:36	5.78
4	B	1	11:08:32 - 11:14:39	6.12
		2	11:17:05 - 11:21:32	4.45
		3	11:35:23 - 11:39:39	4.27

		4	11:42:06 - 11:46:37	4.52
5	B	1	17:12:23 - 17:18:30	6.12
		2	17:20:36 - 17:25:11	4.58
		3	17:26:51 - 17:30:42	3.85
		4	17:42:14 - 17:46:58	4.73
6	B	1	10:36:07 - 10:39:56	3.82
		2	10:41:44 - 10:45:04	3.33
		3	10:47:08 - 10:49:55	2.78
		4	11:50:41 - 11:53:45	3.07
7	B	1	11:12:07 - 11:16:41	4.57
		2	11:19:42 - 11:23:31	3.82
		3	11:25:01 - 11:28:12	3.18
		4	11:31:50 - 11:34:57	3.12
8	B	1	15:40:41 - 15:45:41	5.00
		2	15:47:23 - 15:51:06	3.70
		3	15:53:25 - 15:56:55	3.50
		4	15:58:33 - 16:01:32	2.98
9	B	1	16:20:09 - 16:26:59	6.83
		2	16:29:46 - 16:35:24	5.63
		3	16:58:40 - 17:03:58	5.30
		4	17:05:49 - 17:09:56	4.12

Figure S1. The downward trend of walking time of the blind subjects (Group 2) with the increasing usage count on Route B.

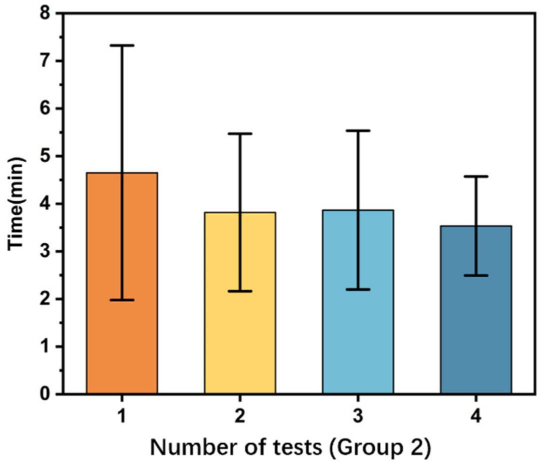

Video S1. Testing for cycling, transportation and stair descending.
